# (Bio)Sensing Strategies Based on Ionic Liquid-Functionalized Carbon Nanocomposites for Pharmaceuticals: Towards Greener Electrochemical Tools

**DOI:** 10.3390/nano12142368

**Published:** 2022-07-11

**Authors:** Álvaro Torrinha, Thiago M. B. F. Oliveira, Francisco W. P. Ribeiro, Pedro de Lima-Neto, Adriana N. Correia, Simone Morais

**Affiliations:** 1 REQUIMTE-LAQV, Instituto Superior de Engenharia do Porto, Instituto Politécnico do Porto, Rua Dr. António Bernardino de Almeida, 431, 4249-015 Porto, Portugal; alvaro.torrinha@graq.isep.ipp.pt; 2 Centro de Ciência e Tecnologia, Universidade Federal do Cariri, Av. Tenente Raimundo Rocha, 1639, Cidade Universitária, Juazeiro do Norte 63048-080, Brazil; thiago.mielle@ufca.edu.br; 3 Instituto de Formação de Educadores, Universidade Federal do Cariri, Rua Olegário Emídio de Araújo, S/N, Centro, Brejo Santo 63260-000, Brazil; wirley.ribeiro@ufca.edu.br; 4 Centro de Ciências, Departamento de Química Analítica e Físico-Química, Universidade Federal do Ceará, Bloco 940, Campus do Pici, Fortaleza 60440-900, Brazil; pln@ufc.br (P.d.L.-N.); adriana@ufc.br (A.N.C.)

**Keywords:** ionic liquid, carbon nanomaterials, electrochemical sensors, pharmaceuticals, electroanalysis

## Abstract

The interaction of carbon-based nanomaterials and ionic liquids (ILs) has been thoroughly exploited for diverse electroanalytical solutions since the first report in 2003. This combination, either through covalent or non-covalent functionalization, takes advantage of the unique characteristics inherent to each material, resulting in synergistic effects that are conferred to the electrochemical (bio)sensing system. From one side, carbon nanomaterials offer miniaturization capacity with enhanced electron transfer rates at a reduced cost, whereas from the other side, ILs contribute as ecological dispersing media for the nanostructures, improving conductivity and biocompatibility. The present review focuses on the use of this interesting type of nanocomposites for the development of (bio)sensors specifically for pharmaceutical detection, with emphasis on the analytical (bio)sensing features. The literature search displayed the conjugation of more than 20 different ILs and several carbon nanomaterials (MWCNT, SWCNT, graphene, carbon nanofibers, fullerene, and carbon quantum dots, among others) that were applied for a large set (about 60) of pharmaceutical compounds. This great variability causes a straightforward comparison between sensors to be a challenging task. Undoubtedly, electrochemical sensors based on the conjugation of carbon nanomaterials with ILs can potentially be established as sustainable analytical tools and viable alternatives to more traditional methods, especially concerning in situ environmental analysis.

## 1. Introduction

**Figure 1:**
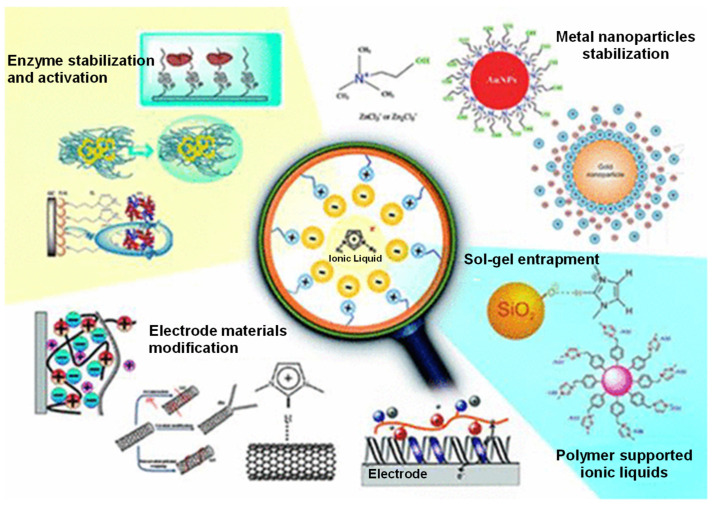
(Bio)technological applications of ionic liquid-based electrochemical systems. Reprinted from Ghorbanizamani and Timur [5]. Copyright 2018—American Chemical Society.

Until the late 20th century, our knowledge of low-melting organic salts was very limited [1]. It is believed that the first literary records arose from the realization that AlCl_3_-catalyzed Friedel-Crafts acylation of toluene generated a red-colored oil phase as a by-product, characterized by nuclear magnetic resonance spectroscopy as a liquefied heptachlorodialuminate salt [2]. This special class of compounds was defined as ionic liquids (ILs), i.e., neoteric solvents evolved from traditional inorganic salts, which are liquids at temperatures ≤100 °C [2,3]. They are typically engineered from bulky organic cation weakly paired with (in)organic anions, and this size asymmetry reduces the Coulomb attractions between counterions, preventing crystallization of the resulting molecular structures [3,4]. In addition to low melting points, ILs have negligible volatility, high polarity, and electrical conductivity. Modulating these properties by tuning ion composition and size makes ILs suitable for challenging (bio)technological applications in which traditional molecular liquids are incompatible [3]. In the electrochemistry field, some sophisticated and innovative ideas are compiled in Figure 1.

**Figure 2:**
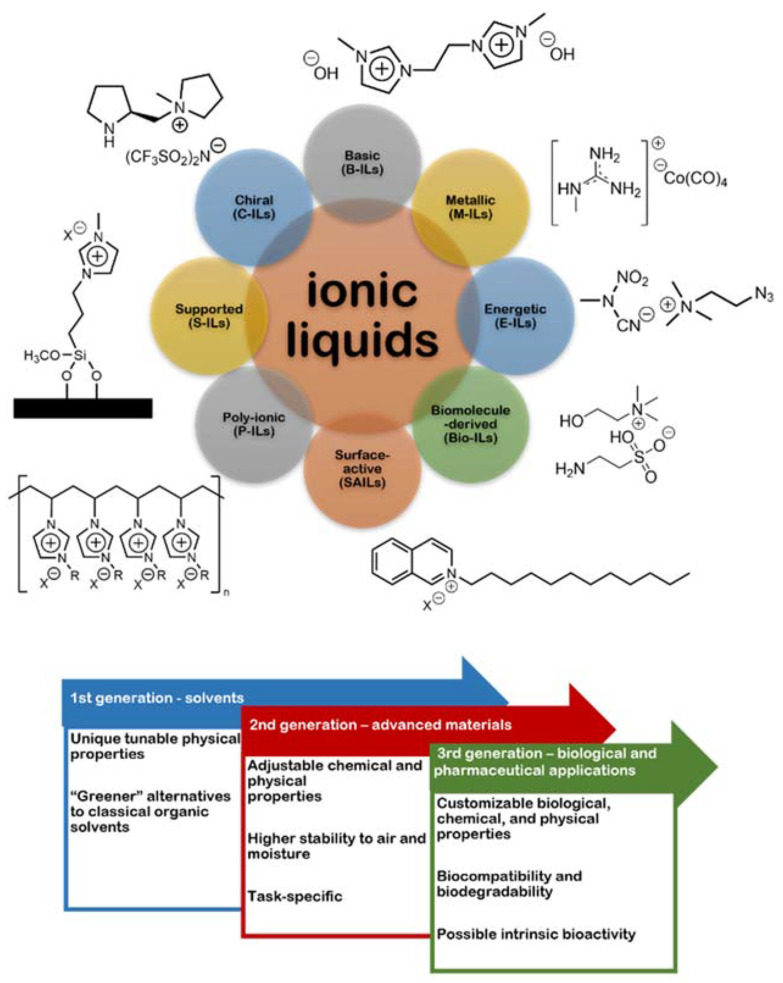
Examples of ionic liquids (ILs) from different generations, along with their properties and uses. Adapted from Gomes et al. [13]. Copyright 2021—MDPI, under Creative Commons Attribution license.

The electrochemical properties of ILs had already provoked curiosity since the first literary record published by Paul Walden in 1914 [6,7], reporting on the peculiar conductive features of ethylammonium nitrate as a “molten salt” (12 °C melting point), though without the current repercussions. The fast diffusion of ILs in this and other areas has accompanied the improvement in their functionalities over the years. Pioneering research with neoteric solvent prototypes composed of cations derived from dialkylimidazole and pyridine, paired with AlCl_4_^−^ or other metal halides, has led to the discovery of first-generation ILs (water- and air-sensitive compounds). After the 1980s, new formulations were designed from halides and other bulkier anions, such as BF_4_^−^, PF_6_^−^ and CH_3_OSO_3_^−^, which remain weakly coordinated to cations and reach moderate polarity, increasing inertia to moisture and air—second-generation ILs. Currently, these last ones are among the most investigated in applied electrochemistry, especially as substitutes for ordinary polar organic solvents that inactivate biological macromolecules [8,9,10]. With the urgent need to develop eco-friendly materials and technologies, third-generation ILs have emerged, preserving the features of the previous generation but innovating with biodegradable, potentially recyclable, and lower toxicity cations (e.g., choline, amino acids, and imidazoles) and/or anions (e.g., saccharinate, alkylsulfates, and alkylphosphates) [4,10,11]. Some authors also include deep eutectic solvents (DESs; Lewis or Brønsted acid-base mixtures, which may contain cationic and/or anionic species) within this ILs category [4], but others prefer to study them as a new class of advanced solvents [12]. The contradiction relies on the fact that DESs may also contain uncharged hydrogen-bond donors (e.g., urea, oxalic acid, and glycerol), so they are not fully ionic. Examples, properties, and uses of ILs from different generations can be seen in Figure 2.

The improvement in ILs over the generations made it possible to develop compounds with electrical conductivity (0.1–20 mS [14]) and electrochemical stability (potential window ≤ 5.0 V [15,16]) uncommon in conventional electrolytes. Consequently, the electrochemical applications of these materials have grown dynamically and intensely in recent decades, showing usefulness in electrosynthesis [17], electrocatalysis [15], CO_2_ capture and reduction [18,19], solid-state electrolytes [17], corrosion inhibitors [20], batteries, supercapacitors, fuel cells, (bio)sensors [3,7,10], among other cutting-edge technology systems. For electrochemical (bio)sensors, important advances have been made in designing, developing, and implementing devices and methods that reduce or eliminate hazardous chemicals in the environment. Among the 12 principles of green chemistry described by Paul Anastas and John Warner in 1998, electrochemical systems involving ILs fulfill at least four of them [21,22,23]: (i) safer solvents—principle no. 5; (ii) suit energy efficiency systems—principle no. 6; (iii) induce electrocatalysis—principle no. 9; and (iv) provide inherently safer chemistry for accident prevention—principle no. 12.

**Figure 3:**
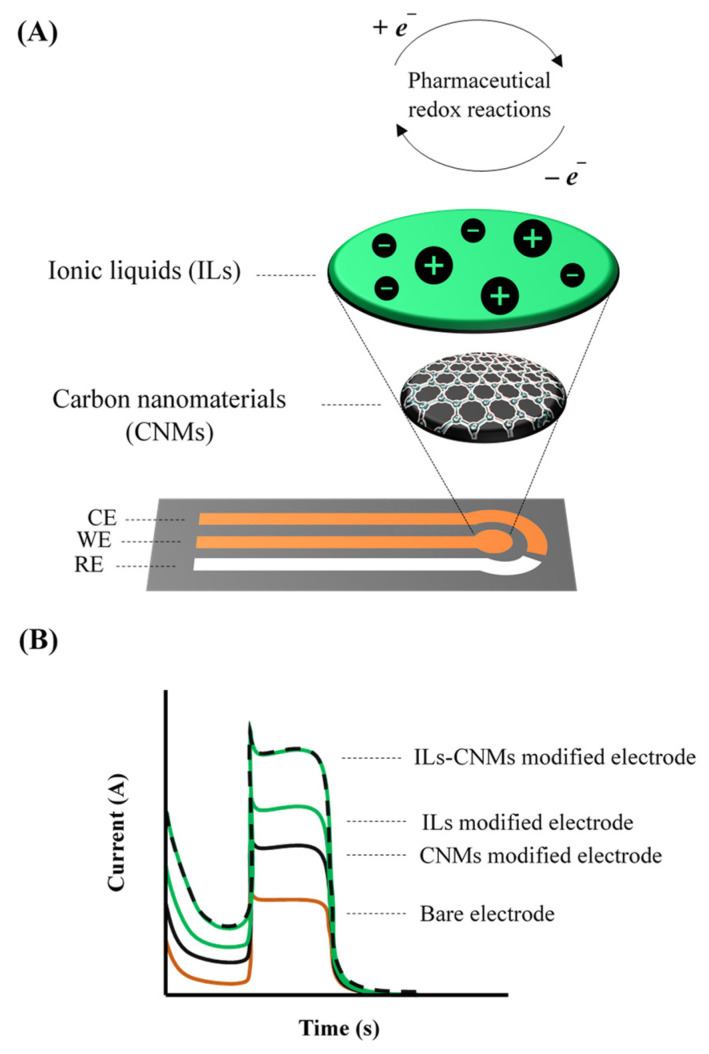
(**A**) Simplified assembly scheme of electrochemical sensors based on ionic liquid-functionalized carbon nanocomposites, along with the drug detection principle. (**B**) Matching electrochemical signal.

Sophisticated (bio)sensors have been proposed by introducing ILs into nanocomposites; casting and adsorbing them on bare or modified electrode surfaces; or transferring by electrodeposition, layer-by-layer, sol-gel encapsulation, and sandwich-type systems [8,16]. In all cases, ILs advantages were harnessed in association with different semiconductors, especially nanostructured carbon allotropes, such as nanotubes (CNT) and graphene [24]. Such nanomaterials provide greater conductivity, electroactive area, electrocatalytic effect, mechanical resistance, and chemical stability to the devices, expanding their application spectrum [24,25,26,27]. The synergism and versatility of IL-based carbon nanocomposites offer promising electroanalytical opportunities for numerous emerging contaminants and bioactive molecules [28,29,30] and, in this review, a snapshot of the most recent findings, with pharmaceutical compounds being an important class of contaminants to be taken in consideration. A general outline of the assembly, detection principle, and analytical signal recorded with these electrochemical sensors is shown in Figure 3. These analytical approaches are relevant for applications in health sciences and also environmental health since the unintentional release of pharmaceuticals (ng L^−1^ to µg L^−1^) into the environment can trigger adverse effects on aquatic ecosystems and human health [27]. Technological limitations in conventional effluent treatment exacerbate contamination rates, as they are inefficient in removing recalcitrant compounds, in addition to enabling the retransformation of metabolites into their precursors or even more toxic derivatives-negative wastewater mass balances [31,32,33]. Diffusion of pharmaceuticals through the environment also facilitates ingestion and absorption by the surrounding fauna, potentially leading to bioaccumulation and biomagnification [34]. Clinical and environmental analyses have been mostly performed through hyphenated methods, especially conjugating chromatography and mass spectrometry or spectrophotometry, since they enable multi-residues analysis with high selectivity and suitable sensitivity [35,36]. However, electrochemical (bio)sensors based on IL-functionalized carbon (CNT, graphene, among others) nanocomposites constitute a more viable option when practicability, speed, operational cost, portability, and sustainable practices are also required.

The literature on electrochemical (bio)sensors comprising the combination of ILs and nanomaterials is very rich. Therefore, the present review will focus specifically on carbon-based nanomaterials due to their efficiency, availability, and importance to the electroanalytical field. Here we give an overview of the characteristics of electrochemical (bio)sensors composed of the different carbon (nano)materials (MWCNT, SWCNT, graphene, carbon nanofibers, carbon black, carbon quantum dots, mesoporous carbon, and fullerene) functionalized with ILs, addressing the analytical performance for comparison purposes. As far as we are concerned, this is the first review on the electrochemical detection of pharmaceuticals by carbon nanomaterials-IL nanocomposites-based (bio)sensors. A total of 78 studies were found on this specific subject and concerns all the available data retrieved from the Web of Science database with no restrictions on time period.

## 2. (Bio)Sensing Strategies for Pharmaceuticals Using Electrochemical Devices Assembled with IL-Functionalized Carbon Nanocomposites

### 2.1. Carbon Nanotubes-IL Nanocomposites

CNT in the form of individual (SWCNT) or multiple concentric tubes (MWCNT) is probably the most employed nanomaterial in electroanalytical applications, mainly attributed to their mechanical, thermal, electronic, and electrocatalytic properties, as well as availability and controllable synthesis. These unique properties are dependent on the structural tube dimensions as well as the helical nature of the tube defined by the chiral vector and chiral angle [37,38]. These chirality factors greatly influence the electronic properties of CNT, presenting a semi-conductive to metallic behavior [37,39,40]. Regarding the electrocatalytic activity of CNT, this can be attributed either to edge-plane sites and defect areas rich in oxygen functionalities or to metallic impurities present in the structure that result from the synthesis process [41,42]. In addition, the high surface area conferred by CNT highly impacts the electrochemical signal of the system, yet, this is only efficiently achieved if CNT is well dispersed in the matrix and not entangled, as they have a tendency to aggregate [24]. Normally this problem is minimized through the use of organic solvents such as N,N-dimethylformamide, though the inherent solvent toxicity forms relatively stable nanotube dispersions though with some inherent toxicity. Alternatively, IL is effectively employed in the dispersion of CNT either alone or in aqueous/organic solutions. This converges in IL-CNT hybrids that present a synergistic effect in terms of, for example, high electric and ionic conductivities. The interaction between IL and CNT can be non-covalent (based on cation-π and/or π-π conjugations) or through covalent functionalization with the aid of coupling agents. This last method produces more stable composites but possibly affects mechanical and electronic properties [24].

#### 2.1.1. MWCNT-IL Nanocomposites

This interesting hybrid nanocomposite formed by CNT and ILs has been extensively applied in the development of electrochemical sensors for pharmaceuticals detection, with MWCNT being largely considered over SWCNT. A total of 42 works were identified in the literature comprising the combination of at least MWCNT and an IL, with the characteristics of the developed sensors and their sensing features presented in Table 1.

Of the 39 different pharmaceutical compounds under study, the majority concern anti-inflammatory and analgesic-type drugs, probably explained by their frequent consumption, known electrochemistry, and low price, being ideal drugs for sensor validation. When considering the anti-inflammatory drug diclofenac, four different ILs were used in combination with MWCNT, which likely contribute to different sensing performances. In this regard, sensors comprising 1-butyl-3-methylimidazolium hexafluorophosphate (BMIM.PF_6_) obtained better limits of detection (LOD). For instance, a carbon ceramic electrode (CCE) made by a sol-gel method was simply modified by dropcast with the mixture of the IL, BMIM.PF_6_ and MWCNT in DMF/water achieved a sensitivity diclofenac of 0.2 μA μmol^−1^ L and a LOD of 0.027 μmol L^−1^ [44]. An equal sensor (CCE/MWCNT-IL) revealed better analytical performance to diclofenac in terms of LOD (0.018 μmol L^−1^) and sensitivity (0.41 μA μmol^−1^ L), even with the detection occurring simultaneously with other anti-inflammatory drugs, namely indomethacin [45]. In addition, a modified carbon paste (CP) sensor using in the mixture of the same IL, BMIM.PF_6,_ alongside MWCNT, graphite was applied for the determination of diclofenac through square wave voltammetry (SWV), obtaining a LOD of 0.09 μmol L^−1^, with authors registering better performance in comparison with a traditional CP electrode, besides a lower drug oxidation potential in 70 mV [46]. In turn, using a slightly different IL in the composite mixture of the CPE, by changing the cation to N-hexyl-3-methylimidazolium (HMIM.PF_6_), the obtained LOD was about two times higher (0.2 μmol L^−1^) than the previously described CPE sensor; however, differential pulse voltammetry (DPV) was employed in the drug analysis instead SWV [47]. In the CPE sensor proposed by Damiri et al. [48], carboxylic acid-functionalized MWCNT containing cobalt hexacyanoferrate nanoparticles (MWCNT-CoHFc) were first synthesized and then mixed with the IL 1-butyl-3-methylimidazolium chloride (BMIM.Cl) and liquid paraffin. Despite applying a well-nanostructured sensor for diclofenac detection, it did not translate into better performance; in fact, this CPE sensor presented the highest LOD value (0.3 μmol L^−1^) for this drug [48] (Table 1).

Besides diclofenac, the pharmaceutical drug acetaminophen was also considerably studied with MWCNT-IL nanocomposites containing different ILs. In this regard, functionalized MWCNT (dispersed in the polymer Nafion) and IL (BMIM.PF_6_) were thoroughly optimized to a ratio of 1:5 with the mixture being deposited in a cleaned glassy carbon electrode (GCE). In optimized conditions, this sensor exhibited a high sensitivity of 2.09 μA μmol^−1^ L and a LOD of 0.067 μmol L^−1^ [49]. A few years earlier, MWCNT was instead dispersed in a matrix of chitosan and then mixed with the IL, 1-ethyl-3-methylimidazolium tetrafluoroborate (EMIM.BF_4_) to give a 5% (*V/V*) concentration, followed by deposition also in a GCE. The sensor was successfully applied for simultaneous detection of four different compounds (acetaminophen, mefenamic acid, ascorbic acid, and uric acid), which probably contributed to lower performance in terms of sensitivity (0.325 μA μmol^−1^ L) and LOD (0.24 μmol L^−1^) for acetaminophen [50]. The same IL (EMIM.BF_4_) was applied for non-covalent modification of MWCNT that was previously decorated with amino-functionalized iron oxide nanoparticles (MWCNT-Fe_3_O_4_(NH_2_)-IL). The nanostructured GCE revealed a remarkable sensitivity toward acetaminophen of 102 μA μmol^−1^ L with a corresponding LOD of 0.04 μmol L^−1^ [51]. For acetaminophen, the lowest LOD (0.003 μmol L^−1^) was achieved by a sensor containing the piridinium-based IL, 1-hexyl-pyridinium hexafluorophosphate (HPy.PF_6_); however, the efficiency of the modified CPE sensor is attributed probably more to the use of titanium oxide nanoparticles (TiO_2_) in the mixture in addition to the MWCNTs [52].

The analysis of analgesic opioid drugs has also been considered, although in less extent. In this respect, the same research group has studied the drug morphine with two different ILs, namely HMIM.PF_6_ and BMIM.Cl. In the first case, a CPE sensor was fabricated with an optimized amount of MWCNT (20% *w*/*w*) and IL (15.5% *w*/*w*), with the remaining percentage being graphite (64.5% *w*/*w*). In optimum conditions and through the DPV technique, morphine could be detected in a wide linear range (0.6–600 μmol L^−1^), with the sensor achieving a LOD of 0.02 μmol L^−1^ [54]. In the following study, the CPE sensor also contained nickel oxide nanoparticles that were synthesized on MWCNT being then mixed in a paste alongside BMIM.Cl, graphite, and paraffin. This further nanostructuration of the CPE led to a decrease in LOD value by half (0.01 μmol L^−1^). The detection of morphine was also tested in the presence of diclofenac, showing well resolved SWV peaks for both drugs, with the sensor practically maintaining the same sensitivity toward morphine when analyzed individually (about 0.05 μA μmol^−1^ L) [55]. The proposal from Atta et al. [56] consisted of a CPE sensor fabricated through the mixing of the IL, 1-butyl-1-methylpiperidinium hexafluorophosphate (BMPy.PF_6_) (25% *w*/*w*), MWCNT (15% *w*/*w*), cobalt oxide nanoparticles (Co_3_O_4_) (2% *w*/*w*), graphite (58% *w*/*w*) and paraffin for simultaneous detection nalbuphine and tramadol in urine. In this medium, the sensor achieved excellent results represented by a sensitivity of about 0.49 μA μmol^−1^ L and LODs of 0.00058 μmol L^−1^ for nalbuphine and 0.00062 μmol L^−1^ for tramadol when both analyzed by DPV in the presence of the surfactant sodium dodecyl sulfate (SDS) [56].

**Figure 4:**
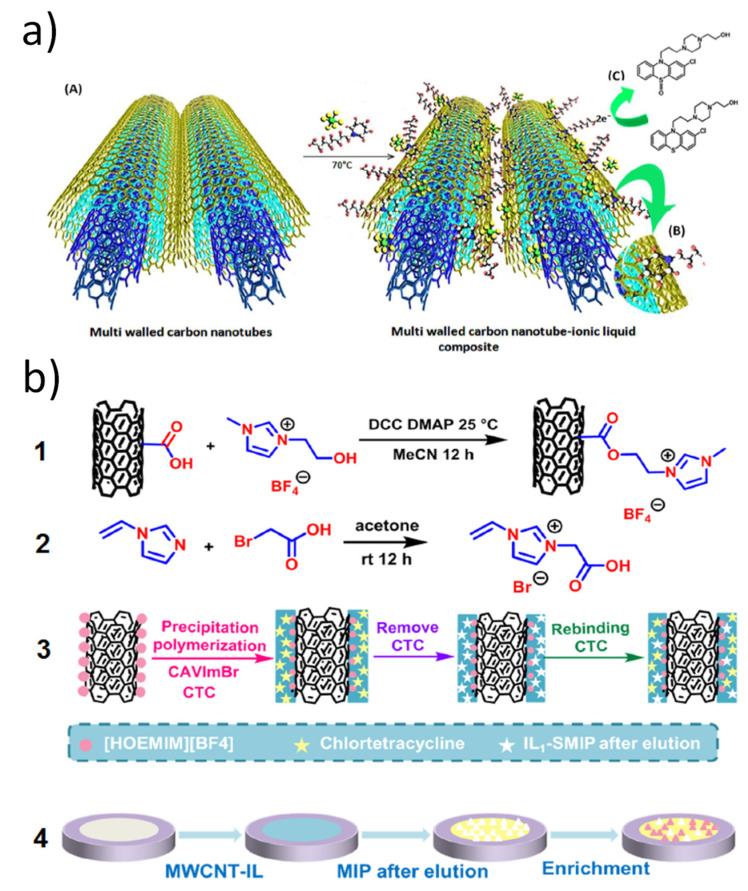
(**a**) Scheme representing dispersion of MWCNT in OPy.PF_6_ (**A**), the cation-π interaction (**B**), and oxidation of perphenazine drug (**C**) (reproduced from Fasihi et al. [59], with permission from the Royal Society of Chemistry, 2015). (**b**) Scheme of a MIP-based chlortetracycline sensor representing MWCNT modification by IL, HEMIM.BF_4_ (1), synthesis of 1-carboxymethyl-3-vinylimidazolium bromide (2), preparation of MIP at the MWCNT-IL surface (3), and further modification of a GCE (4) (reproduced from Chen et al. [66], with permission from Elsevier, 2021).

Several antidepressive and antipsychotic drugs were also subject of study from MWCNT-IL nanocomposite-based sensors, although the variability of analyzed drugs impedes a more strictly comparison between the developed sensors. Briefly, for this group of drugs, eight different compounds were analyzed by sensors that comprised three different ILs in their composition. Various paste-based sensors employed the IL n-octylpyridinum hexafluorophosphate (OPy.PF_6_) in the mixture apart from MWCNT and graphite, obtaining a more or less concordant analytical performance in terms of LOD, varying from 0.0065 μmol L^−1^ for risperidone [57] drug to 0.023 μmol L^−1^ for perphenazine (Figure 4a) [59]. The cation-π interaction of carbon nanotubes with the IL, OPy.PF_6_ and their application for oxidation of perphenazine is well demonstrated in Figure 4a. Recently, the frequently used IL, BMIM.PF_6_ was employed in a sensor for simultaneous detection of clozapine and sertraline, though the dispersion of MWCNT occurred also with the aid of DMF and not exclusively with the IL. After modification of a GCE, further nanostructuration occurred through electrodeposition of nickel oxide nanoparticles at a fixed potential of -0.8 V. Under DPV analysis at an optimum pH of 7, the sensor presented similar results for both drugs with linear range, sensitivity, and LOD of 0.5 to 67 μmol L^−1^, 0.52 μA μmol^−1^ L, and 0.052 μmol L^−1^, respectively, for clozapine and 0.21 to 85 μmol L^−1^, 0.53 μA μmol^−1^ L, and 0.047 μmol L^−1^, respectively for sertraline [61]. In a different approach, Tarahomi et al. [62] applied both IL and MWCNT in an electrode modification intended for carbamazepine though not promoting a direct mixture between these two materials in a composite. Specifically, a CPE was first assembled by mixing graphite, paraffin, and the IL 1-butyl-3-methylimidazolium bis (trifluoromethylsulfonyl) imide (BMIM.TSFI). Its surface was then modified by dropcast with the composite mixture of MWCNT and previously synthesized lanthanum nanorods (LaNRs), followed by a final film of Nafion. Although laborious, the sensor reached a low LOD of 0.006 μmol L^−1^ in optimum acidic conditions (pH 2), also revealing great stability after 4 weeks by maintaining 98% of the response [62].

Antibiotics and antiviral drugs are another group of pharmaceuticals subject to significant study by electrochemical sensors, given their wide consumption and posed risks to the environment and human health. Still, concerning specifically MWCNT-IL nanocomposite-based electrochemical sensors, only four studies were identified in the literature and were focused on pharmaceuticals with less expression. As an example, the direct-acting antiviral daclatasvir, a novel drug used to treat the hepatitis C virus, was determined in serum samples by a GCE modified with different layers. The sequence consisted in applying first a layer of DMF-dispersed MWCNT, followed by BMPy.PF_6_ in DMF, a second deposition of MWCNT, and a final layer of iron oxide nanoparticles (Fe_3_O_4_). By DPV analysis in pH 2, this sensor registered two linear zones from 0.003 to 0.1 μmol L^−1^ and from 0.5 to 15 μmol L^−1^, accomplishing a high sensitivity of 154 μA μmol^−1^ L and a very low LOD of 4 × 10^−5^ μmol L^−1^ with these results being determined in diluted serum medium [64]. Suitable results have also been achieved in the simultaneous sensing of tuberculosis-treating drugs, ethambutol (sensitivity of 17.4 μA μmol^−1^ L and LOD of 0.02 μmol L^−1^) and pyrazinamide (sensitivity of 13.4 μA μmol^−1^ L and LOD of 0.01 μmol L^−1^). The configuration of this sensor consisted of the modification of a GCE with a layer of MWCNT decorated with cobalt ferrite nanoparticles (CoFe_2_O_4_). Interaction between MWCNT and IL occurred further through dipping the modified GCE into EMIM.BF_4_ for 90 min followed by 10 min at 4 °C and finally dried at room temperature for 5 min. An interesting sensing approach was adopted by Chen et al. [66] by using molecularly imprinted polymer (MIP) technology based on IL monomer to greatly enhance the selectivity of the antibiotic chlortetracycline. Also noteworthy is the fact that MWCNT was covalently functionalized with IL instead of the frequently employed strategy of non-covalent interaction. Briefly, the functionalization occurred through a reaction between carboxylic acid-functionalized MWCNT, the IL, 1-hydroxyethyl-3-methylimidazolium tetrafluoroborate (HEMIM.BF_4_), the coupling agent, N,N′-dicyclohexylcarbodiimide (DCC) and 4-(dimethylamino) pyridine (DMAP). This functionalized MWCNT-IL nanocomposite was dispersed in DMF and deposited on the surface of a GCE followed by deposition of the MIP consisting in IL 1-carboxymethyl-3-vinylimidazolium bromide as functional monomer, ethylene glycol dimethacrylate as crosslinker and the drug as template (Figure 4b). The LOD achieved corresponded to 0.08 μmol L^−1^ although the sensor stands out for its selectivity when tested with several analogous compounds.

The presence of hormones in the environment is always a subject of great concern due to their disruptive effects. However, only one study has performed validation of a sensor comprised of an MWCNT-IL (BMIM.PF_6_)-modified GCE specifically in environmental samples (river water), reaching recoveries between 97% and 105% [67], whereas the other considered hormones, namely norepinephrine [68,69] and epinephrine [70] were related to clinical analysis. In this regard, CPE sensors composed of a mixture of BMIM.Br, MWCNT, graphite, and paraffin obtained practically the same results either for norepinephrine [68] or for epinephrine [70]. The additional incorporation of ZnO nanoparticles into MWCNT and conjugation with the IL 1,3-dipropylimidazolium bromide (dPIM.Br) managed to lower the LOD by about 78% for norepinephrine [69]. A similar CPE sensor as the one just described but using HMIM.PF_6_ instead was developed by the same research group for the detection of carbidopa, an antiparkinson drug. The analysis performed by SWV at optimum pH of 6 resulted in a wide linear range detection (0.09–450 μmol L^−1^), reaching a LOD of 0.05 μmol L^−1^ [72]. Previously, the simpler CPE sensor (containing graphite, paraffin, MWCNT, and BMIM.Br) proposed by Beitollah et al. [71] did not differ significantly in terms of performance for carbidopa, also obtaining a wide linear range from 0.1 to 420 μmol L^−1^ and similar LOD (0.06 μmol L^−1^) but lower sensitivity (0.028 μA μmol^−1^ L).

According to Table 1, the vast majority of the works employed either DPV or SWV technique in drug determinations due to their higher sensitivity and discrimination capacity when compared with cyclic voltammetry and amperometry, respectively. In this sense, Norouzi et al. [75] further enhanced SWV sensitivity by introducing a fast Fourier transform where the signal is calculated based on admittance changes related to the changes in the electrical double layer. The coupling of this technique with a GCE-based sensor comprising a first nanocomposite layer of MWCNT and IL (EMIM.BF_4_) dispersed in DMF and a second layer of electrosynthesized gold nanoparticles (AuNPs) enabled obtaining a very low LOD for amlodipine, corresponding to 1.25 × 10^−4^ μmol L^−1^ [75]. Conversely, the amperometry technique drawback is the lack of discrimination capacity when applied for multi-analyte detection or applied in samples containing other electroactive compounds besides the analyte of interest. Therefore, only one study has applied it as a determination technique, particularly for sulfadiazine antibiotics. In this case, MWCNT and OPy.PF_6_ were simply mixed (1:9 w/w), dispersed in DMF, and deposited in a clean GCE, being applied for drug detection in strongly acidic conditions at +0.9 V, presenting a LOD of 0.21 μmol L^−1^. The selectivity parameter was tested with ascorbic acid, and since it oxidized at a lower potential (+0.35 V) compared to the sulfadiazine, its interference was eliminated by signal subtraction [63]. Linear sweep voltammetry (LSV) is another technique employed, however limitedly. Besides the previously described work from Tao et al. [67] regarding the detection of the hormone estradiol through LSV, other two works have used this technique, specifically for the antihypertensive drug nitrendipine [76] and antihistamine drug chlorpheniramine [81]. Another aspect remarkably observable in Table 1 is the almost exclusive use of CPE and GCE-type electrodes that serve as modification substrates for MWCNT-IL nanocomposites. The exception is the work of Wang et al. who fabricated a screen-printed electrode composed by the mixture of cellulose, OPy.PF_6_ and graphite and further modified by chitosan dispersed MWCNT. The sensor was applied for rutin detection attaining a sensitivity of 0.78 μA μmol^−1^ L and a LOD of 0.02 μmol L^−1^, but also suitable reproducibility (<5% RSD), which validated its application in real samples [84].

It is worth mentioning the lack of biosensors that could exploit the synergistic effect of MWCNT-IL nanocomposites on bioelectrocatalysis. The sole example regards the development of an aptasensor for an ibuprofen anti-inflammatory drug (Table 1). This biosensor consisted of the modification of a GCE by a composite mixture involving MWCNT, IL 1-methyl-3-octylimidazolium tetrafluoroborate (MOIM.BF_4_), and chitosan, followed by sequential deposition of terephthalaldehyde, capure probe (ssDNA1), ibuprofen-specific aptamer (ssDNA2) and methylene blue (MB). As expected, the biorecognition of ibuprofen was accomplished with high sensitivity (0.77 μA [log(pmol)]^−1^ L) and a LOD of 2 × 10^−5^ μmol L^−1^ [53]. The authors attributed these excellent results not only to the MWCNT-IL nanocomposite, which increased conductivity and surface area, but also to the use of chitosan, which is rich in amine groups that lead to an increase in the amount of immobilized aptamers as well as to the specificity of the ibuprofen-aptamer reaction. 

#### 2.1.2. SWCNT-IL Nanocomposites

Nanocomposites enriched with ILs and SWCNT have multifunctional characteristics in electrochemical sensors designed for pharmaceuticals. As previously stated, studies involving IL-SWCNT nanocomposites are less found in the literature compared to IL-MWCNT, probably explained by the higher price of SWCNT, even though the first report on the interaction between CNTs and ILs was with SWCNT [86]. Only nine studies have specifically addressed the application of SWCNT-IL nanocomposites for the detection of a total of nine different pharmaceuticals (Table 2). The IL EMIM.BF_4_ was the most used in conjunction with SWCNT, with this nanocomposite being applied for epinephrine (also known as adrenaline, an important sympathomimetic hormone, and neurotransmitter that works against Parkinson’s disease) and the analgesic drug acetaminophen. In this regard, Ayazi et al. [87] proposed a carbon ceramic electrode simply modified with a thin film of a composite that consisted of SWCNT (3 mg) and EMIM.BF_4_ (60 μL) for detection of epinephrine in the presence of uric acid. Immidazolium-based ILs can interact with CNT via π-π pairing, and this assists in the control of van der Waals interactions between nanostructures, as well as in the homogeneity and organization of the nanocomposite at the sensor interface. The sensor reached high selectivity when analyzing epinephrine in the presence of common interferents such as ascorbic acid, uric acid, dopamine, and acetaminophen, as well as suitable sensitivity through a low LOD of 0.028 μmol L^−1^, being ultimately validated in human serum and urine samples. In the other proposed sensor for epinephrine, also applied simultaneously to acetaminophen, the same IL (EMIM.BF_4_) was used and mixed with chitosan dispersed SWCNT at an optimized concentration of 6% (v_IL_/v_total_), being further casted in a GCE [88]. The results were slightly worse for epinephrine in terms of LOD by obtaining a three times higher value (0.09 μmol L^−1^) compared with the previously described sensor [87], probably due to some electrical resistance caused by chit polymer, whereas, for acetaminophen, the sensitivity corresponded to 0.847 μA μmol^−1^ L and LOD to 0.06 μmol L^−1^ [88]. Exactly the same sensor configuration was used by the same authors in a study regarding the simultaneous determination of acetaminophen, ascorbic acid, and uric acid by DPV [89]. The feasibility of the sensor in the determination of the three compounds was demonstrated through well-separated peaks though this conclusion was somewhat evident in the interferent assay in their last study [88]. The few studies on SWCNT-IL nanocomposite and the variability of analyzed pharmaceuticals (Table 2) prevent a more straightforward comparison between sensors. To highlight the contribution of Xiao et al. [90], who studied the reduction peak of the antibiotic drug chloramphenicol by testing different ILs in the hybrid film also composed of SWCNT and AuNPs. The signal-to-noise ratio depended on the IL type: 1-octyl-3-methylimidazolium hexafluorophosphate (OMIM.PF_6_) > BMIM.PF_6_ > BMIM.BF_4_ > BMIM.Br, with this effect being attributed to the greater hydrophobicity of OMIM.PF_6_, which facilitates interfacial drug accumulation. Therefore, this IL was selected for further investigations in drug detection, with the sensor reaching a LOD of 0.005 μmol L^−1^. The authors found out that IL was essential to improve film uniformity and stability, avoiding material loss and secondary residue formation, besides enlarging the device’s electroactive area. The IL at volume ratios ≤ 2.0% also increased the analyte accumulation at the sensor interface and detection sensitivity, but above this value, the charge-transfer was difficult [90]. Also noticeable is the CPE sensor for daunorubicin (anticancer) developed by Alizadeh et al. [91] that registered a very wide linear range, from 0.008 to 350 μmol L^−1^ and the lowest LOD value (0.003 μmol L^−1^) from the SWCNT-IL-based nanocomposites presented in Table 2. However, this performance was accomplished with a very well-nanostructured CPE that involved the mixing of the IL 1-butyl-2,3-dimethylimidazolium tetrafluoroborate (BdMIM.BF_4_) with SWCNT previously modified with NiO, Pt and Pd nanoparticles. All the sensors present in Table 2 were validated in clinical field type samples with exception of chloramphenicol sensor, which was validated in milk samples.

### 2.2. Graphene-Based IL Nanocomposites

#### 2.2.1. Combination of Graphene and MWCNT in the IL Nanocomposite

The 2D carbon nanomaterial such as graphene (rich in sp^2^-hybridized carbon atoms) is one of the main sensing agents that have been applied in modern electroanalytical chemistry for the determination of several classes of pharmaceutical compounds due to its unique properties. As demonstrated by several studies, graphene is a versatile material for (bio)sensor design due to its ability to form nanocomposites with several other materials. Composites based on IL combined with both MWCNT and graphene have been applied for the determination of pharmaceutical compounds. The MWCNT-graphene hybrid has the ability to form π-π interaction between these two materials, resulting in the enhancement of the surface area, which consequently improves the electrochemical signal and thus the sensitivity. The addition of IL can fill the voids between the CNT and graphene layers.

**Figure 5:**
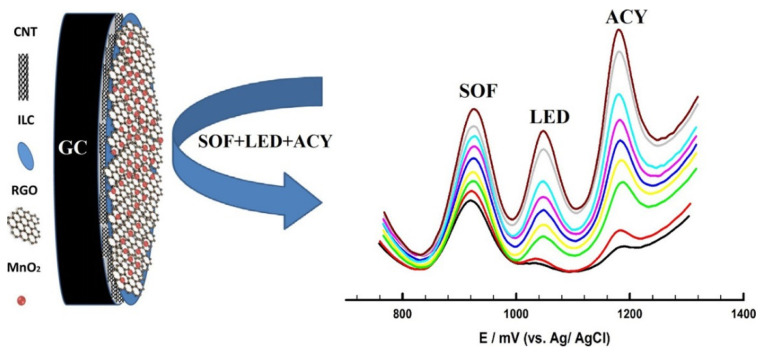
General scheme of GCE/CNT/ILC/RGO/MnO_2_ sensor development and electroanalytical signal related to the simultaneous oxidation of antiviral drugs sofosbuvir (SOF), ledipasvir (LED), and acyclovir (ACY) (reproduced from Atta et al. [96], with permission from Elsevier, 2014).

As far as we know, only four studies reported the use of MWCNT-graphene-ILs nanocomposites that were applied in the sensing of eight different pharmaceuticals in diverse types of samples of the clinical (pharmaceutical formulations, blood plasma, and serum), environmental (lake and pond water) and food (pork) areas (Table 3). In this type of nanocomposite, emphasis is given to both sensors developed by Atta’s group [96,97] for effective simultaneous analysis. In their follow-up study [64], reduced graphene oxide (rGO) was additionally used in a layer-by-layer modification procedure of a GCE. Basically, the sensor consisted of the first deposition of MWCNT (dispersed in DMF), then deposition of BMPip.PF_6_ (in DMF) followed by electrochemical reduction in GO and a final electropolymerization of 18-Crown-6. The simultaneous detection of three drugs, namely acetaminophen, amlodipine, and dobutamine, was successfully accomplished with very low LOD [97]. A very similar sensor for the determination of antiviral drugs (sofosbuvir, ledipasvir, and acyclovir), but instead electrodeposited MnO_2_ nanoparticles as the last layer (Figure 5), was developed [96]. Although these two developed sensors [96,97] were based on more laborious modification procedures compared with most sensors described here, they were very efficient in achieving subnanomolar levels of detection (LODs in the order of 10^−4^ µmol L^−1^). Also undertaking considerable efforts on sensor modification was the study conducted by Chen et al. [98], in line with the previously described MIP sensor for chlortetracycline [66]. In this proposal, the sensor for oxytetracycline antibiotics consisted of a first modification step with a nanocomposite of IL-modified nitrogen-doped rGO and MWCNT. This nanocomposite was interestingly formed through the mixture of GO solution with MWCNT, urea, and the IL, HEMIM.BF_4_ in a 50 mL Teflon-lined autoclave and subjected to a hydrothermal process. The nanocomposite was collected, dispersed in DMF, and applied to the GCE surface. Next, carbon-silica composite was synthesized, mixed with HAuCl_4_ for the formation of AuNPs on the surface and further carbonized to produce gold modified carbon nanospheres composite (Au-CNS). This composite was used as a support for surface imprinting using the ILs, methyl 2-(3-vinylimidazolidin-1-yl) acetate bromide and 1,6-di (3-vinylimidazolium) hexane bromide, as functional monomer and cross-linking agent, respectively, for MIP synthesis, with the drug serving as a template. The nanocomposite MIP was finally deposited on the previously modified electrode (GCE/N_rGO-MWCNT-IL/Au-CNS-IL-MIP). As a consequence of the high nanostructuration level and use of MIP, the sensor was both selective and sensitive (LOD of 0.005 µmol L^−1^) to oxytetracycline. In addition, it also presented suitable reproducibility (6.6% RSD), repeatability (5.4% RSD) and storage stability (maintaining 93% of the response after 4 weeks). This sensor was ultimately intended for assessment of the drug levels in contaminated food products (pork) and environmental waters (lake and pond waters) [98].

#### 2.2.2. Other Graphene-Based IL Nanocomposites

A total of 12 sensors based on other graphene-IL nanocomposites were developed for the determination of 15 different pharmaceuticals assessed in matrices of pharmaceutical formulations, serum, and urine samples. Antibiotics were the class of drugs most analyzed by nanocomposites based on graphene and IL, representing six different sensors (Table 4). In this regard, Peng et al. [100] demonstrated that a simple modification of a GCE with BMIM.PF_6_-graphene composite (dispersed in DMF) was efficient in the determination of azithromycin antibiotics in pharmaceutical formulations. In addition, another antibiotic (metronidazole) was investigated by a similar sensor but using chitosan additionally as dispersing matrix of the nanocomposite, with the sensor reaching a LOD of 0.047 µmol L^−1^ [101]. A related IL, namely BMIM.BF_4_, was, in turn, mixed with graphene oxide (GO), graphite, and paraffin to produce a very sensitive CPE-based sensor for ofloxacin detection (LOD of 2.8 × 10^−4^ and sensitivity of 7.7 µA µmol^−1^ L) [102]. The authors evaluated the electron transfer rate constant, k^o^, through electrochemical impedance spectroscopy analysis for different electrodes (CPE, CPE with GO, CPE with IL, and IL-GO containing CPE). The results showed the enhancement of the electron transfer kinetics for the modified electrode containing IL and GO. In addition, another similar IL but with the anion BF_4_ substituted by Br (BMIM.Br) was effectively used in a CPE for sensing sulfamethoxazole antibiotic in pharmaceutical formulations and urine [103]. 

**Figure 6:**
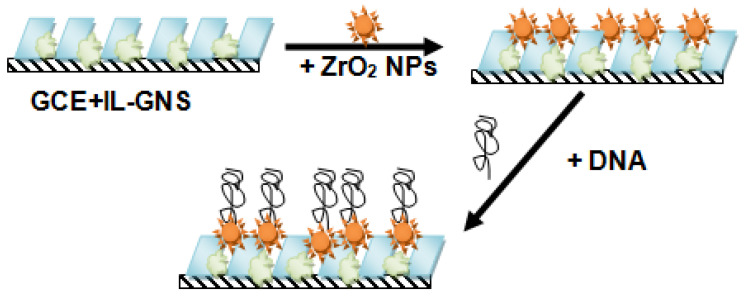
Preparation of the biosensor based on calf thymus DNA immobilized on a GCE previously modified with graphene nanosheets (GNS), 1-butyl-3-methylimidazolium hexafluorophosphate ionic liquid (BMIMPF_6_), and ZrO_2_ nanoparticles for the detection of rutin (reproduced from Norouzi et al. [110], with permission from International Journal of Electrochemical Science).

Other pharmaceutical compounds from different classes such as levodopa, cabergoline, methocarbamol, rutin (Figure 6), and raloxifene have been the subject of study by IL-graphene nanocomposites. It is worth highlighting the sensor developed by Atta et al. [109], who managed to detect the muscle relaxant drug, methocarbamol with high sensitivity (3.02 μA μmol^−1^ L), reaching a very low LOD of 6.64 × 10^−6^ μmol L^–1^. This GCE-based sensor consisted of the application by dropcast of different layers of the unusual IL, BMPip.PF_6_ and rGO and a final layer of cyclodextrin. According to the authors, the moderate viscosity of IL penetrates between the graphene sheets, filling any existing voids and acting as ion carriers between the graphene sheets, which results in a more conductive composite.

### 2.3. Other Carbon-Based IL Nanocomposites

Other carbon (nano)materials, namely carbon nanofibers (CNF), carbon black (CB), mesoporous carbon, carbon quantum dots (CQD), and fullerene, have been conjugated with IL and applied for pharmaceuticals detection. Likewise seen for MWCNT-IL nanocomposite, the IL BMIM.PF_6_ was the most employed in the nanocomposite mixture, whereas anti-inflammatory and analgesic drugs were the most studied by this type of sensor (Table 5). Among them, carbon nanofibers (CNF) stand out for the preparation of new sensors since they are cheaper than graphene and carbon nanotubes [112,113,114]. The same research group has been characterizing the preparation of CNF-based nanocomposites using different ILs [112,113,114]. In the first example, a carbon paste was formed by mixing BMIM.PF_6_ with CNF that was previously modified with polyaniline (PANI) and AuNPs was successfully applied in the determination of naproxen anti-inflammatory by DPV [112]. In a sequent study, Afzali et al. [114] described an electroanalytical procedure based on DPV for the determination of the anti-inflammatory drug colchicine using a GCE modified with cupric oxide nanoparticles, CNF, and 1-butyl-3-methylimidazolium tetrachloroferrate (BMIM.FeCl_4_). In addition, Afzali et al. [113] proposed an electroanalytical methodology based on SWV for the determination of pemetrexed, an anticancer drug, using a CPE modified with palladium nanoparticle (Pd), CNF, and a newly synthesized IL, methyl (trioctyl)ammonium bis(trifluoromethylsulfonyl)imide (M3OA.NTF2), and Nafion with this last component being used as a binder. All the proposed sensors by Afzali et al. [112,113,114] remarkably achieved high sensitivities (34.5 µA µmol^−1^ L for colchicine to 3904 µA µmol^−1^ L for naproxen). The last study concerns the anchoring of AuNPs on graphitized CNF combined with BMIM.PF_6_ and carbon paste to produce a highly sensitive (49 µA µmol^−1^ L) and selective determination of the anticancer drug irinotecan [115]. The CNF-based IL nanocomposites revealed to be very efficient for detection of the respective drugs, though the downsize is that metallic nanoparticles were also used, which prevents a proper assessment of CNF-IL nanocomposite role on analytical outcome.

Carbon materials such as mesoporous carbon or CB are hardly seen as nanomaterials due to their micrometer size; nevertheless, these were considered in the scope of the present review since they can present similar behavior regarding the fabrication of electrochemical sensors. A sensor prepared from the mixture of highly defective mesoporous carbon (DMC), the common IL, BMIM.PF_6_, besides graphite and mineral oil, was successfully applied to establish an electroanalytical methodology for the quantification of rutin, an antioxidant compound in *Ruta graveolens* extract, pharmaceutical tablets, and orange juice [118].

Carbon black is obtained from the partial combustion or thermal decomposition of gaseous or liquid hydrocarbons; because of the higher electrical conductivity, high specific surface area, particle size, and stability, this material has numerous applications, including coatings. For the purpose of diclofenac detection, Cunha et al. [116] developed a sensor based on CB and BMIM.PF_6_ matrix to modify the surface of pencil graphite electrodes (PGE). The voltametric profiles displayed an anodic peak at 0.8 V and a second anodic peak at 0.55 V, as related to the first one, being attributed to the amine oxidation. The modified PGE sensor achieved a low value of LOD (0.08 µmol L^−1^), and it was validated in tablets. Fathirad et al. [117] used a nanofiber-based composite formed by Pd nanoparticles loaded on Vulcan CB, IL, and Nafion to determine tramadol in pharmaceutical and biological samples (tablet, healthy human urine, infected human urine, and human blood plasma). The IL monomer based on 1-methylimidazole was polymerized in the presence of azobis (2-methylpropionitrile) and 1 wt% PdVC in ethanol. This study reported a synergistic improvement in the electrical conductivity of the as-prepared sensor.

Newer carbon materials such as carbon quantum dots combined with IL have also been the subject of study. These are carbonaceous materials (mainly in the graphene-type sp^2^ hybridization, with amounts of the diamond-type sp^3^ hybridization) composed of amorphous to crystalline carbon base displaying excellent optical absorptivity, unique optical properties, chemical inertness, and facile synthesis, being applied, for instance, in medical diagnosis, and chemical sensing. Concerning this approach, Sanati and Faridbod [119] successfully developed a sensor using graphene quantum dots and BMIM.PF_6_ on CPE for methyldopa determination in tablets and serum. In addition, a CPE sensor for analysis of raloxifene in the presence of tamoxifen was developed by mixing nitrogen-doped carbon quantum dots (N_CQD) on Fe_3_O_4_ nanoparticles, BMIM.BF_4_, graphite, and paraffin [120]. The detection through DPV occurred at about +0.5 V (vs. Ag/AgCl) for both drugs with two well-defined peaks, achieving linear concentration range and LOD values corresponding, respectively, to 0.04–320 μmol L^−1^ and 0.01 μmol L^−1^ for raloxifene.

Fullerene C_60_, which is a carbon allotrope that presents a closed structure with a ball shape, was also used in a nanocomposite alongside MWCNT and BMIM.BF_4_ for the determination of diazepam in real samples, including serum, urine, and tablets. The experimental results confirmed that the modified electrode with C_60_-MWCNT-IL has suitable electrocatalytic activity toward the reduction in diazepam. The electrocatalytic current increases linearly with the diazepam concentration in the ranges of 0.3–700.0 µmol L^−1^, and the achieved LOD corresponded to 0.087 µmol L^−1^ [121].

## 3. Discussion

Different conjugations of carbon nanomaterials and ILs dictate the development of electrochemical sensors with different particularities in the detection of pharmaceutical compounds. Comparison of efficiency and analytical performance between these sensors for a given compound is a challenging exercise since many factors influence the electrochemical response. Besides sensor configuration and nanostructuration, which are probably the most impacting, other factors such as electrode material, electrode pre-treatment, the employed electrochemical technique, and medium pH are also highly relevant to accomplish satisfactory sensitivity, selectivity, reproducibility, and stability. Ideally, a correct comparison between different carbon nanomaterials conjugated with ILs should be performed for the same pharmaceutical and similar sensor configuration. For instance, acetaminophen has been analyzed by different carbon nanomaterials-based IL nanocomposites involving other materials with varied configurations. However, a fairer comparison can be made for sensors based on the use of EMIM.BF_4_ and chitosan in the composite but using either MWCNT [50] or SWCNT [88,89], with the latter achieving slightly better analytical performance in terms of sensitivity and LOD. In turn, diclofenac analysis by similar modification procedures using Vulcan CB or MWCNT [44,45,46] conjugated with the same IL, BMIM.PF_6_ produced sensors with LOD values in the same order of magnitude independently of the type of electrodes used, either CCE [44,45], CPE [46], or PGE [116]. In fact, the proposal of Cunha et al. [116] is a suitable example that the choice of cheap materials such as the pencil mine-based transducer or the “nanomaterial” CB may be an interesting alternative to more expensive nanomaterials such as MWCNT.

On the other hand, a significant difference is verified in sensors for methyldopa detection, where a CPE composed of graphene quantum dots [119] achieved 10 times lower LOD than a CPE with MWCNT in its composition [74], though with different IL employed (BMIM.PF_6_ in the former sensor [119] and BMIM.Br in the later [74]). In a general overview, it is difficult to assess significant variations in terms of the sensor’s analytical performance when comparing MWCNT-IL, SWCNT-IL, and graphene-IL nanocomposites, represented respectively in Table 1, Table 2, Table 3 and Table 4. Without a doubt, MWCNT-IL nanocomposite has been vastly studied in pharmaceutical detection over other carbon-based nanocomposites, possibly due in part to lower market cost and lower processability requirements if not acid-treated. In the case of graphene, it may require a reduction in the oxygen functionalities, which may lead to higher capacitive currents, especially when electrochemically reduced. However, sensors developed on the basis of CNF-IL nanocomposite achieved very high sensitivity (>20 µA µmol^−1^ L) and nanomolar to subnanomolar LOD [112,113,114,115] though this nanocomposite group is not significantly represented (only four sensors) and additionally employed metallic nanoparticles in the composite mixture (Table 5).

ILs are considered biocompatible and sustainable solvents due to the absence of vapor pressure, constituting an advantage over organic solvents such as acetonitrile and DMF [122]. However, these apolar organic solvents, especially DMF, have been widely employed in the dispersion of the carbon nanomaterial alone [44,45,49,82,99,114], the IL nanocomposite [63,75,81,98], or even the IL itself [64,97] since certain ILs are solid crystals at room temperature [24]. Conversely, other studies employed ILs directly as a dispersing agent of the carbon nanomaterial [53,67,76,83,113,116], exploiting the advantages that were first observed by Fukushima et al. [24]. The dispersing ability and the viscosity and sticky nature of ILs enable the formation of pastes that are preferably used in CPEs, being the most employed type of sensor (about 54% of total sensors) in the analytical determination of pharmaceuticals. Despite the suitable biocompatibility offered by carbon nanomaterial-IL-based composites, only two studies have adopted a biosensing strategy in the detection of pharmaceuticals and were specifically based on the immobilization of DNA [53,110].

The type of samples used in sensor validation is also noticeable. It seems that most of the developed sensors were intended for the clinical field since they were tested or validated in samples of human blood serum, human urine, and pharmaceutical formulations that typically contain the studied drug-like tablets, injections, oral solutions, etc. The presence of pharmaceuticals in the environment is a growing problem for aquatic species but also humans. In this sense, only eight studies have validated the proposed sensing approaches in environmental or food type samples [43,53,66,67,72,74,90,98]. In part, this is explained in many cases by the absence of the required sensitivity as the concentration of pharmaceutical compounds present in this type of sample are usually on the nanomolar level or below. Relevant examples are the works of Roushani et al. [53] and Chen et al. [98], who detected ibuprofen and oxytetracycline in wastewater and river water in levels reaching 10^−5^ and 10^−3^ μmol L^−1^, respectively, although employing complex, time consuming, or expensive procedures in order to achieve those levels.

## 4. Conclusions

The literature on the conjugation of carbon-based nanomaterials and ILs into interesting and synergistic nanocomposites is very rich, and this is clearly patented on their specific application in the detection of pharmaceutical compounds. A total of 78 electrochemical sensors comprising carbon nanomaterial-IL nanocomposites were found in the literature. From these, further discrimination translated into the use of 23 different ILs, conjugated with 8 types of carbon (nano)materials, namely MWCNT, SWCNT, graphene, CNF, CB, mesoporous carbon, fullerene, and carbon quantum dots. This expresses the versatility and the number of possibilities and opportunities that exist in the development of new electrochemical (bio)sensing systems. Despite ILs being considered more ecologically sustainable dispersing agents, forming stable interactions with carbon nanomaterials, their sole use for this purpose is still limited and usually disregarded by DMF or other equivalent organic solvents.

Also obvious is the difficulty in assessing specific contribution and the efficiency of the carbon nanomaterial-IL nanocomposite for the electrochemical response of a pharmaceutical given the wide spectrum of compounds analyzed as well as variability in employed ILs, besides other factors. In this respect, only two works have equated and compared different ILs in the nanocomposite mixture for pharmaceutical detection in the same study. Thus, additional research is clearly needed to explore in a more comprehensive way the greener and synergistic properties of IL-functionalized carbon nanocomposites for the design of more ecological and efficient (bio)sensors for pharmaceuticals analysis.

Furthermore, the almost exclusive use of GCE- and CPE-based sensors clearly evidences a lack of investment in miniaturized and fully integrated electroanalytical solutions that can be suitably used for point-of-care diagnostics or in situ environmental analysis. Another critical fact is the limited number of studies that validated the developed (bio)sensors in environmental-type samples, which means that the environmental problem that pharmaceutical compounds take part in is still significantly neglected by the scientific community. This is also supported by the limited studies on, e.g., hormones that are frequently found in the environment and are highly toxic due to endocrine-disruptive effects in the aquatic species. In our view, taking care of the environment is taking care of ourselves; thus, increasing investment in environmental solutions should be adopted, with electroanalysis potentially having a crucial role in preventive and control measures.

## Figures and Tables

**Table 1:** Electrochemical sensors based on MWCNT-IL nanocomposites for pharmaceutical compounds.

Analyte	Ionic Liquid, IL	(Bio)Sensor	Detection Technique	Linear Range (μmol L^−1^)	Sensitivity (μA μmol^−1^ L)	LOD (μmol L^−1^)	Real Samples	Ref.
**Anti-Inflammatories/Analgesics**								
diclofenac	EMIM.PF_6_	GCE/Cu(OH)_2_-MWCNT-IL-paraffin	DPV	0.18–119	0.0147	0.04	fish serum seawater pharm. formul.	[43]
diclofenac	BMIM.PF_6_	CCE/MWCNT-IL	DPV	0.05–20	0.2	0.027	plasma	[44]
diclofenac	BMIM.PF_6_	CCE/MWCNT-IL	DPV	0.05–50	0.406	0.018	pharm. formul. plasma	[45]
indomethacin				1–50	0.24	0.26		
diclofenac	BMIM.PF_6_	CPE(graphite + MWCNT + paraffin + IL)	SWV	0.3–35 35–750	0.1 0.029	0.09	pharm. formul. urine	[46]
diclofenac	HMIM.PF_6_	CPE(graphite + MWCNT + IL + paraffin)	DPV	0.5–300	-	0.2	pharm. formul. urine	[47]
diclofenac	BMIM.Cl	CPE(MWCNT-CoHCF + IL + paraffin)	DPV	1–100	0.208	0.3	pharm. formul. urine	[48]
acetaminophen	BMIM.PF_6_	GCE/MWCNT-Nafion-IL	SWV	0.3–3	2.09	0.067	pharm. formul.	[49]
acetaminophen	EMIM.BF_4_	GCE/MWCNT-IL-chit	DPV	1–400	0.325	0.24	serum urine	[50]
mefenamic acid				2–650	0.116	1.2		
acetaminophen	EMIM.BF_4_	GCE/MWCNT-Fe_3_O_4_(NH_2_)-IL	DPV	0.01–0.7	102	0.04	pharm. formul.	[51]
acetaminophen	HPy.PF_6_	CPE(graphite + IL + MWCNT + TiO_2_)	SWV	0.01–30	1.05	0.003	plasma pharm. formul.	[52]
ibuprofen	MOIM.BF_4_	GCE/MWCNT-Chit-IL/terephthalaldehyde/ssDNA1/ssDNA2/MB	DPV	7 × 10^−5^–6	7.7 × 10^5^	2 × 10^−5^	pharm. formul. serum wastewater	[53]
morphine	HMIM.PF_6_	CPE(graphite + paraffin + MWCNT + IL)	DPV	0.6–10 10–600	0.15 0.019	0.02	pharm. formul. urine	[54]
morphine	BMIM.Cl	CPE(graphite + paraffin + NiO-MWCNT + IL)	SWV	0.05–520	0.0521	0.01	pharm. formul. urine	[55]
nalbuphine	BMPip.PF_6_	CPE(graphite + paraffin + IL + MWCNT + Co_3_O_4_)	DPV	0.06–10	0.49	5.8 × 10^−4^	urine pharm. formul.	[56]
tramadol				0.06–10	0.486	6.2 × 10^−4^		
**Antidepressive/Antipsychotic**								
risperidone	OPy.PF_6_	CPE(graphite + MWCNT + IL)	DPV	0.01–0.2	16	0.0065	pharm. formul. serum	[57]
diazepam	OPy.PF_6_	CPE(graphite + MWCNT + IL)	SWV	0.07–2.7	6.8	0.012	pharm. formul. serum urine	[58]
Oxazepam				0.17–6.6	0.66	0.02		
perphenazine	OPy.PF_6_	CPE(graphite + MWCNT + IL)	DPV	0.05–30 30–150	2.41 0.55	0.023	pharm. formul. serum	[59]
amitriptyline	OPy.PF_6_	CPE(graphite + MWCNT + IL)	DPV	0.05–90	0.3723	0.019	pharm. formul.	[60]
clozapine	BMIM.PF_6_	GCE/MWCNT-IL/NiO	DPV	0.5–67	0.5146	0.052	pharm. formul. serum	[61]
sertraline				0.21–85	0.5306	0.047		
carbamazepine	BMIM.TFSI	CPE(graphite + paraffin + IL)/LaNR-MWCNT/Nafion	SWV	0.06–20	0.02	0.006	pharm. formul. urine	[62]
**Antibiotic**								
sulfadiazine	OPy.PF_6_	GCE/IL-MWCNT	amperometry	3.3–35.4	0.214	0.21	pharm. formul.	[63]
daclatasvir	BMPip.PF_6_	GCE/MWCNT/IL/MWCNT/ Fe_3_O_4_	DPV	0.003–0.1 0.5–15	154	4 × 10^−5^	serum pharm. formul.	[64]
ethambutol	EMIM.BF_4_	GCE/MWCNT-CoFe_2_O_4_/IL	DPV	0.2–2.2	17.37	0.02	pharm. formul.	[65]
pyrazinamide				0.6–2.8	13.66	0.01		
chlortetracycline	HEMIM.BF_4_	GCE/MWCNT-IL/IL-MIP	DPV	0.4–10 10–55	2.58 1.32	0.08	pharm. formul. milk tap water	[66]
**Hormone**								
estradiol	BMIM.PF_6_	GCE/MWCNT-IL	LSV	0.01–1 1–7.5	30.58 6.29	0.005	river water serum	[67]
norepinephrine	BMIM.Br	CPE(graphite + paraffin + MWCNT + IL)	DPV	0.3–30 30–450	0.0841 0.0231	0.09	pharm. formul. urine serum	[68]
norepinephrine	dPIM.Br	CPE(graphite + paraffin + IL + ZnO-MWCNT)	SWV	0.05–8 8–450	2.946 0.349	0.02	pharm. formul. urine	[69]
epinephrine	BMIM.Br	CPE(graphite + paraffin + MWCNT + IL)	DPV	0.3–450	0.0237	0.09	pharm. formul. serum urine	[70]
**Antiparkinson**								
carbidopa	BMIM.Br	CPE(graphite + paraffin + MWCNT + IL)	SWV	0.1–110 110–420	0.028 0.014	0.06	serum urine	[71]
carbidopa	HMIM.PF_6_	CPE(graphite + paraffin + MWCNT-ZnO + IL)	SWV	0.09–3.5 3.5–450	0.986 0.109	0.05	serum urine water	[72]
levodopa	BPy.PF_6_	CPE(graphite + paraffin + Fe_3_O_4_-SiO_2_-MWCNT + IL + PHC)	DPV	0.06–20 20–400	0.294 0.0294	0.02	pharm. formul. serum urine	[73]
Cabergoline				0.07–350	0.08	0.019		
**Antihypertensive**								
methyldopa	BMIM.Br	CPE(graphite + paraffin + MWCNT + IL)	SWV	0.4–400	2.78	0.1	urine pharm. formul. water	[74]
amlodipine	EMIM.BF_4_	GCE/MWCNT-IL/AuNPs	CDFFTAV	0.001–0.2	-	1.25 × 10^−4^	pharm. formul.	[75]
nitrendipine	BMIM.PF_6_	GCE/MWCNT-chit-IL	LSV	0.4–50	0.77	0.1	pharm. formul.	[76]
**Others**								
alfuzosin	HPy.PF_6_	CPE(graphite + IL + MWCNT)	DPV	0.02–90	0.635	0.0041	plasma	[77]
sulfasalazine	HMIM.PF_6_	CPE(graphite + NiO-MWCNT + IL)	SWV	0.5–800	0.046	0.09	pharm. formul. urine	[78]
folic acid	dPIM.Br	CPE(graphite + paraffin + IL + MWCNT-ZnO)	SWV	0.08–650	-	0.05	pharm. formul. mint leaves juice	[79]
L-tryptophan	BMIM.PF_6_	CPE(graphite + paraffin + IL + Pt-MWCNT)	SWV	0.1–400	0.0469	0.04	pharm. formul. meat extract	[80]
chlorpheniramine	BMIM.BF_4_	GCE/MWCNT-IL	LSV	1–90	-	0.7	pharm. formul.	[81]
pseudoephedrine	BMIM.PF_6_	GCE/MWCNT/MWCNT-IL	DPV	240–980	0.104	196	pharm. formul.	[82]
chlorpheniramine				1.4–100	0.84	0.42		
ciprofibrate	BMIM.Cl	GCE/DHP-MWCNT-IL	DPV	0.25–7.41	-	0.092	pharm. formul.	[83]
rutin	OPy.PF_6_	SPE/cellulose-IL-graphite/chit-MWCNT	DPV	0.05–3.5	0.782	0.02	pharm. formul.	[84]
isoprenaline	BMIM.PF_6_	CPE(graphite + MgO-MWCNT + IL + paraffin)	DPV	6 × 10^−4^–420	-	1 × 10^−4^	pharm. formul.	[85]

AuNPs—gold nanoparticles; CCE—carbon ceramic electrode; CDFFTAV—coulometric differential fast Fourier transformation admittance voltammetry; Chit—chitosan; CoHCF—cobalt hexacyanoferrate; CPE—carbon paste electrode; DHP—dihexadecylphosphate; DPV—differential pulse voltammetry; GCE—glassy carbon electrode; IL—ionic liquid; LaNR—lantanium nanorods; LSV—linear sweep voltammetry; MB—methylene blue; MIP—molecularly imprinted polymer; MWCNT—multi-walled carbon nanotubes; PHC—2-(4-oxo-3-phenyl-3,4-dihydroquinazolinyl)-N’-phenyl-hydrazine-carbothioamide; SPE—screen-printed electrode; SWV—square wave voltammetry. Pharm. formul.—pharmaceuticals formulations (in respect to tablets and/or oral solutions and/or injections).Ionic liquids: BMIM.BF_4_—1-butyl-3-methylimidazolium tetraflouroborate; BMIM.Br—1-butyl-3-methylimidazolium bromide; BMIM.Cl—1-butyl-3-methylimidazolium chloride; BMIM.PF_6_—1-butyl-3-methylimidazolium hexafluorophosphate; BMIM.TFSI—1-butyl-3-methylimidazolium bis (trifluoromethylsulfonyl) imide; BMIM.Br—1-butyl-3-methylimidazolium bromide; BMPip.PF_6_—1-butyl-1-methylpiperidinium hexafluoro phosphate; BPy.PF_6_—N-butylpyridinium hexafluorophosphate; dPIM.Br—1,3-dipropylimidazolium bromide; EMIM.BF_4_—1-ethyl-3-methylimidazolium tetrafluoroborate; EMIM.PF_6_—1-ethyl-3-methylimidazolium hexafluorophosphate; HEMIM.BF_4_—1-(2-hydroxyethyl)-3-methylimidazolium; HMIM.PF_6_—N-hexyl-3-methylimidazolium hexafluorophosphate; HPy.PF_6_ - 1-hexyl-pyridinium hexafluorophosphate; MOIM.BF_4_—1-methyl-3-octylimidazolium tetrafluoroborate; OPy.PF_6_—n-octylpyridinum hexafluorophosphate.

**Table 2:** Electrochemical sensors developed from SWCNT-IL-based nanocomposites for pharmaceutical analysis.

Analyte	Ionic Liquid, IL	Sensor	Detection Technique	Linear Range (µmol L^−1^)	Sensitivity (µA µmol^−1^ L)	LOD (µmol L^−1^)	Real Sample	Ref.
diphenhydramine	BMIM.PF_6_	CPE(SWCNT-CdO + IL)	SWV	0.05–700	0.163	0.009	pharm. formul. serum	[92]
raloxifene	BMPy.BF_4_	CPE(graphite + SWCNT-NiO + paraffin + IL)	SWV	0.03–520	0.158	0.007	pharm. formul. serum	[93]
mycophenolate	HMIM.PF_6_	CPE(graphite + SWCNT-MgO + IL + paraffin)	SWV	0.1–450	0.031	0.07	pharm. formul. serum	[94]
chloramphenicol	OMIM.PF_6_	GCE/AuNPs-SWCNT-IL	DPV	0.01–6	0.532	0.05	milk	[90]
epinephrine	EMIM.BF4	CCE/SWCNT-IL	DPV	0.1–200	0.376	0.028	serum urine	[87]
epinephrine acetaminophen	EMIM.BF4	GCE/SWCNT-chit-IL	DPV	1–580 0.5–400	0.500 0.847	0.09 0.06	serum urine	[88]
acetaminophen	EMIM.BF4	GCE/SWCNT-chit-IL	DPV	2–200	0.328	0.11	urine serum	[89]
daunorubicin	BdMIM.BF_4_	CPE(graphite + Pt-Pd-NiO-SWCNT + IL + paraffin)	DPV	0.008–350	0.227	0.003	pharm. formul. dextrose serum	[91]
adrenalone folic acid	BMIM.MS	CPE(graphite + SWCNT-NiO + IL + paraffin)	DPV	0.001–400 0.3–350	0.193 0.279	0.006 0.07	pharm. formul.	[95]

AuNPs—gold nanoparticles; CCE—carbon ceramic electrode; Chit—chitosan; CPE—carbon paste electrode; DPV—differential pulse voltammetry; GCE—glassy carbon electrode; SWCNT—single-walled carbon nanotubes; SWV—square wave voltammetry; Pharm. formul.—pharmaceuticals formulations (in respect to tablets and/or oral solutions and/or injections). Ionic liquids: BdMIM.BF_4_—1-butyl-2,3-dimethylimidazolium tetrafluoroborate; BMIM.PF_6_—1-butyl-3-methylimidazolium hexafluorophosphate; BMIM.MS—1-butyl-3-methylimidazolium methanesulfonate; BMPy.BF_4_—1-butyl-4-methylpyridinium tetrafluoroborate; EMIM.BF_4_—1-ethyl-3-methylimidazolium tetrafluoroborate; HMIM.PF_6_—N-hexyl-3-methylimidazolium hexafluoro phosphate; OMIM.PF_6_—1-octyl-3-methylimidazolium hexafluorophosphate.

**Table 3:** Sensors based on a combination of graphene and MWCNT in the IL nanocomposite for pharmaceutical analysis.

Analyte	Ionic Liquid, IL	Sensor	Detection Technique	Linear Range (µmol L^−^^1^)	Sensitivity (µA µmol^−^^1^ L)	LOD (µmol L^−^^1^)	Real Samples	Ref.
naproxen	BMIM.PF_6_	CCE/MWCNT-rGO-IL	DPV	0.8–100	0.3533	0.125	plasma	[99]
acetaminophen amlodipine dobutamine	BMPip. PF_6_	GCE/MWCNT/IL/rGO/CW	DPV	0.001–20 0.008–30 0.02–40	1.81 0.956 0.873	9.06 × 10^−5^ 1.39 × 10^−4^ 4.97 × 10^−4^	pharm. formul. serum	[97]
sofosbuvir ledipasvir acyclovir	BMPip. PF_6_	GCE/MWCNT/IL/rGO/MnO_2_	DPV	0.20–150 0.0070–15 0.010–30	0.049 0.63 0.47	0.0098 1.07 × 10^−4^ 8.43 × 10^−4^	pharm. formul. serum	[96]
oxytetracycline	HEMIM. BF_4_	GCE/MWCNT-N_rGO-IL/Au-CNS-IL-MIP	DPV	0.02–20	2.72	0.005	lake water pond water pork	[98]

CCE—carbon ceramic electrode; CNS—carbon nanospheres; CW—18-crown-6; DPV—differential pulse voltammetry; GCE—glassy carbon electrode; MIP—molecularly imprinted polymer; MWCNT—multi-walled carbon nanotubes; rGO—reduced graphene oxide; Pharm. formul.—pharmaceuticals formulations (in respect to tablets and/or oral solutions and/or injections). Ionic liquids: BMIM.PF_6_—1-butyl-3-methylimidazolium hexafluorophosphate; BMPip.PF6—1-Butyl-1-methylpiperidinium hexafluoro phosphate; HEMIM.BF_4_—1-(2-hydroxyethyl)-3-methylimidazolium tetrafluoroborate.

**Table 4:** Other graphene-IL-based electrochemical sensors for pharmaceutical analysis.

Analyte	Ionic Liquid, IL	(Bio)Sensor	Detection Technique	Linear Range (µmol L^−1^)	Sensitivity (µA µmol^−1^ L)	LOD (µmol L^−1^)	Real Sample	Ref.
**Anti-Inflammatories/Analgesics**								
celecoxib	EMIM.PF_6_	CPE(graphite + rGO + IL + paraffin)/AuNPs	DPV	0.5–15		0.2	pharm. formul. serum	[104]
acetaminophen isoproterenol theophylline	HMIM.PF_6_	CPE(graphite + GrNS + BBFT + IL + paraffin)	SWV	10–1000 0.06–700 12–1200	0.056 0.731 0.013	8.1 0.012 9.2	pharm. formul. tea serum urine	[105]
**Antibiotics**								
azithromycin	BMIM.PF_6_	GCE/Gr-IL	DPV	0.65–37	-	0.25	pharm. formul.	[100]
metronindazole	BMIM.PF_6_	GCE/Gr-IL-chit	DPV	0.10–25	0.0592	0.047	pharm. formul.	[101]
ofloxacin	BMIM.BF_4_	CPE(graphite + GO + IL + paraffin)	SWAdASV	0.007–0.7	7.7	2.8 × 10^−4^	pharm. formul. urine	[102]
sulfamethoxazole	BMIM.Br	CPE(graphite + paraffin + NiO-GO + IL)	SWV	0.08–550	0.0101	0.04	pharm. formul. urine	[103]
pyrazinamide	EMIM.BF_4_	GCE/AgNPs-rGO/IL	DPV	3–24	0.4547	0.0102	pharm. formul.	[106]
cefixime	EMIM.Cl	CPE(graphite + paraffin + CoFe_2_O_4_-rGO + IL)	DPV	0.06–10 10–700	1.71 0.016	0.035	pharm. formul. urine serum	[107]
**Others**								
levodopa cabergoline	HMIM.PF_6_	CPE(graphite + BBFT + GrNS + IL + paraffin)	SWV	0.05–15 15–800	0.58 0.048	0.015 –	pharm. formul. urine pharm. formul. blood	[108]
methocarbamol	BMPip.PF_6_	GCE/IL/rGO/IL/CD	DPV	0.04–1 8–100	3.015 0.193	6.64 × 10^–6^	urine	[109]
rutin	BMIM.PF_6_	GCE/GrNS-IL/ZrO_2_/DNA	CFFTAV	0.002–0.150	–	2.3 × 10^−4^	pharm. formul.	[110]
raloxifene	dPIM.Br	CPE(graphite + GrNS-ZnO + IL + paraffin)	SWV	0.0001–5 1–500	–	7.0 × 10^–5^	pharm. formul. serum	[111]

AgNPs—silver nanoparticles; BBFT—1-(4-bromobenzyl)-4-ferrocenyl-1H-[1,2,3]-triazole; CD—cyclodextrin; CFFTAV—coulometric fast Fourier transformation admittance voltammetry; CPE—carbon paste electrode; DPV—differential pulse voltammetry; GCE—glassy carbon electrode; GO—graphene oxide; Gr—graphene; GrNS—graphene nanosheets; Rog—reduced graphene oxide; SWV—square wave voltammetry. Pharm. formul.—pharmaceutical formulations (in respect to tablets and/or oral solutions and/or injections). Ionic liquids: BMIM.Br—1-butyl-3-methylimidazolium bromide; BMIM.PF_6_—1-butyl-3-methylimidazolium hexafluorophosphate; BMPip.PF_6_—1-butyl-1-methylpiperidinium hexafluoro phosphate; dPIM.Br—1,3-dipropylimidazolium bromide; EMIM.BF_4_—1-ethyl-3-methylimidazolium tetrafluoroborate; EMIM.Cl—1-ethyl-3-methylimidazolium chloride; HMIM.PF_6_—N-hexyl-3-methylimidazolium hexafluoro phosphate.

**Table 5:** Sensors based on other carbon-based IL nanocomposites for pharmaceutical compounds.

Analyte	Ionic Liquid, IL	Sensor	Detection Technique	Linear Range (µmol L^−1^)	Sensitivity (µA µmol^−1^ L)	LOD (µmol L^−1^)	Real Sample	Ref.
**Anti-Inflammatories/Analgesics**								
naproxen	BMIM.PF_6_	CPE(graphite + CNF-AuNPs-PANI)/IL	DPV	5 × 10^−5^–0.02	3904	1.6 × 10^−5^	pharm. formul. urine	[112]
colchicine	BMIM.FeCl_4_	GCE/CuO-CNF-IL/Nafion	DPV	0.001–0.1	34.5	2.5 × 10^−4^	pharm. formul plasma	[114]
diclofenac	BMIM.PF_6_	PGE/CB-IL	DPV	10–45	-	0.08	pharm. formul.	[116]
tramadol	EIM.VS	GCE/Pd-CB-IL_nanofibers_-Nafion	SWV	0.05–10.0 10.0–200.0	0.812 0.136	0.015	pharm. formul. urine plasma	[117]
**Anticancer**								
pemetrexed	M3OA.NTF2	CPE(graphite + paraffin)/CNF-Pd-IL/Nafion	SWV	0.001–0.035	259	3.3 × 10^−4^	pharm. formul. plasma urine	[113]
Irinitecan	BMIM.PF_6_	CPE(AuNPs-CNF + IL + paraffin)	SWV	0.004–1.79	23.5	0.00155	pharm. formul. serum urine	[115]
**Others**								
rutin	BMIM.PF_6_	CPE(graphite + DMC + IL + paraffin)	SWV	0.008–4	103.7	0.00117	Ruta extract orange juice pharm. formul.	[118]
methyldopa	BMIM.PF_6_	CPE(graphite + paraffin + GQD + IL)	SWV	0.04–750.0	-	0.01	pharm. formul. serum	[119]
raloxifene	BMIM.BF_4_	CPE(graphite + paraffin + N_CQD-Fe_3_O_4_ + IL)	DPV	0.04–320	0.242	0.01	pharm. formul. urine	[120]
diazepam	BMIM.BF_4_	GCE/fullerene-CNT-IL	DPV	0.3–50 50–700	0.173 0.023	0.087	pharm. formul. urine serum	[121]

AuNPs—gold nanoparticles; CB—carbono black; CNF—carbono nanofibers; CPE—carbono paste electrode; DMC—defective mesoporous carbono; DPV—differential pulse voltammetry; GCE—glassy carbono electrode; GQD—graphene quantum dots; N_CQD—nitrogen-doped carbono quantum dots; PANI—polyaniline; PGE—pencil graphite electrode; SWV—square wave voltammetry. Pharm. formul.—pharmaceuticals formulations (in respect to tablets and/or oral solutions and/or injections). Ionic liquids: BMIM.BF_4_—1-butyl-3-methylimidazolium tetraflouroborate; BMIM.PF_6_—1-butyl-3-methylimidazolium hexafluorophosphate; BMIM.FeCl_4_—1-butyl-3-methylimidazolium tetrachloroferrate; EIM.VS—ethylimidazolium vinylsulfonate; M3OA.NTF2—methyl (trioctyl)ammonium bis(trifluoromethylsulfonyl)imide.

## Data Availability

Not applicable.
